# Supplementary material for: Cannabis Use among Cancer Survivors amid the COVID-19 Pandemic: Results from the COVID-19 Cannabis Health Study
Source: Cancers (Basel). 2021 Jul 13;13(14):3495. doi: 10.3390/cancers13143495 (PMC8303109; doi:10.3390/cancers13143495)
Supplement: Supplementary file 1 [file cancers-13-03495-s001.zip › cancers-1285532-supplementary.pdf]

cancers

MDPI

Article

Cannabis Use among Cancer Survivors amid the COVID-19 Pandemic: Results from the COVID-19 Cannabis Health Study

Marlene Camacho-Rivera [1], Jessica Y. Islam [2], Diane L. Rodriguez [3] and Denise C. Vidot [4,*]

1 Department of Community Health Sciences, School of Public Health, SUNY Downstate Health Sciences University, Brooklyn, NY 11203, USA; marlene.camacho-rivera@downstate.edu
2 Cancer Epidemiology Program, H. Lee Moffitt Cancer Center and Research Institute, Tampa, FL 33612, USA; jessica.islam@moffitt.org
3 Morsani College of Medicine, University of South Florida, Tampa, FL 33602, USA; dianer@usf.edu
4 School of Nursing and Health Studies, University of Miami, Coral Gables, FL 33146, USA; dvidot@med.miami.edu
* Correspondence: dvidot@med.miami.edu

Table S1. Substance Use.

	Total		Adults Without Cancer		Cancer Survivors		*p*-value
	No.	Col %	No.	Col %	No.	Col %	
Tobacco Use							0.147
More since COVID-19	10	6.3	4	5.1	6	7.6	
Less Since COVID-19	6	3.8	1	1.3	5	6.3	
No Change since COVID-19	52	32.9	23	29.1	29	36.7	
Never Use	90	57	51	64.6	39	49.4	
Alcohol Use							0.351
More since COVID-19	18	11.5	10	12.8	8	10.3	
Less Since COVID-19	16	10.3	11	14.1	5	6.4	
No Change since COVID-19	74	47.4	36	46.2	38	48.7	
Never Use	48	30.8	21	26.9	27	34.6	
Opioid Use							0.244
More since COVID-19	3	1.9	0	0	3	3.8	
Less Since COVID-19	4	2.6	1	1.3	3	3.8	
No Change since COVID-19	39	25	20	25.6	19	24.4	
Never Use	110	70.5	57	73.1	53	67.9	
Methamphetamine Use							0.632
More since COVID-19							
Less Since COVID-19							
No Change since COVID-19	24	15.7	11	14.3	13	17.1	
Never Use	129	84.3	66	85.7	63	82.9	
Cocaine Use							0.942
More since COVID-19							
Less Since COVID-19	4	2.6	2	2.6	2	2.6	
No Change since COVID-19	34	21.9	18	23.1	16	20.8	
Never Use	117	75.5	58	74.4	59	76.6	
Psilocybin Use							0.101
More since COVID-19	3	2	1	1.3	2	2.7	
Less Since COVID-19	4	2.6	4	5.2	0	0	
No Change since COVID-19	37	24.3	22	28.6	15	20	
Never Use	108	71.1	50	64.9	58	77.3	
